# Blood Pressure Management in Acute Ischemic Stroke

**DOI:** 10.1007/s11906-020-01120-7

**Published:** 2020-12-10

**Authors:** Dariusz Gąsecki, Mariusz Kwarciany, Kamil Kowalczyk, Krzysztof Narkiewicz, Bartosz Karaszewski

**Affiliations:** 1 Department of Adult Neurology, Medical University of Gdańsk, ul, Dębinki 7, 80-952 Gdańsk, Poland; 2 Department of Hypertension and Diabetology, Medical University of Gdańsk, ul, Dębinki 7, 80-952 Gdańsk, Poland

**Keywords:** Blood pressure, Hypertension, Acute ischemic stroke, Management

## Abstract

**Purpose of Review:**

Abrupt blood pressure (BP) rise is the most common clinical symptom of acute ischemic stroke (AIS). However, BP alterations during AIS reflect many diverse mechanisms, both stroke-related and nonspecific epiphenomena, which change over time and across patients. While extremes of BP as well as high BP variability have been related with worse outcomes in observational studies, optimal BP management after AIS remains challenging.

**Recent Findings:**

This review discusses the complexity of the factors linking BP changes to the clinical outcomes of patients with AIS, depending on the treatment strategy and local vessel status and, in particular, the degree of reperfusion achieved. The evidence for possible additional clinical markers, including the presence of arterial hypertension, and comorbid organ dysfunction in individuals with AIS, as informative and helpful factors in therapeutic decision-making concerning BP will be reviewed, as well as recent data on neurovascular monitoring targeting person-specific local cerebral perfusion and metabolic demand, instead of the global traditional parameters (BP among others) alone.

**Summary:**

The individualization of BP management protocols based on a complex evaluation of the homeostatic response to focal cerebral ischemia, including but not limited to BP changes, may be a valuable novel goal proposed in AIS, but further trials are warranted.

## Introduction

Stroke is globally a leading cause of mortality and long-term disability in adults. Even though the prevalence and death rate due to stroke have decreased over the last years, the overall burden remains high, and the absolute number of people affected continues to increase [1, 2]. Acute ischemic stroke (AIS) accounts for approximately 70–85% of all strokes worldwide.

AIS is caused by a critical reduction in blood flow to the brain. Cerebral blood flow (CBF) is almost totally arrested in the core region of the insult, which leads to neuronal death within minutes. The tissue surrounding the core is severely hypoperfused and functionally impaired but still viable. This zone, called the penumbra, is at high risk of infarction. It permits cell survival for a certain period of time; however, it is extremely vulnerable to CBF fluctuations, which depend on both local and global factors, such as cerebral vasoreactivity or collaterals and systemic components, especially blood pressure (BP). Therefore, the salvation of the ischemic penumbra is the holy grail of vascular neurology. There are two fundamental factors which allow this aim: (i) early recanalization and reperfusion and (ii) the maintenance of CBF before reperfusion occurs.

Strategies aiming at fast reperfusion, including intravenous thrombolysis (IVT) and mechanical thrombectomy (MT) within 4.5 and 6 h (in selected patients, up to 24), respectively, from the stroke onset, remain the most effective therapeutic methods in standard care. However, the proper management of multiple clinical problems other than—or secondary to—arterial occlusion is also significantly associated with the final outcome. In this regard, hemodynamics plays a special role.

BP is one of the most important modifiers of vascular function and organ perfusion, and its abnormalities are related to vascular dysfunction, with cerebral circulation appearing as one of the most susceptible to BP dysregulations.

Ischemic stroke is a consequence of a variety of pathological conditions, resulting from either systemic cardiovascular (CV) diseases or primary cerebrovascular diseases, which, in the great majority, may be considered as hypertensive or hypertension related. Therefore, a large proportion of AIS is a consequence of chronic hypertension (HT).

On the other hand, acute BP elevation is one of the most common phenomena observed in the first hours and days after AIS onset, which potentially strongly influences clinical decisions and modifies the risk of acute complications.

There is a growing body of evidence that BP may be an important factor related to stroke outcome; however, the available evidence shows conflicting results. Indeed, BP elevation after stroke, being a common aspect of many processes, is rather descriptive, if not an epiphenomenon of a plethora of influencing factors, and further data is needed to predict stroke outcome.

We believe that the associations of BP in the acute phase of stroke with its clinical course should be analyzed as part of a long-standing process, which might begin even many years before the event, rather than as an acute phenomenon occurring only in the first days after the disease onset. It seems to us that a paradigm shift in the management of BP in AIS is needed: from simplistic, based just on sole BP values, to more complex, including the identification and management of compensatory mechanisms, modified by chronic diseases, especially HT and, what is even more relevant, adjusted to local hemodynamic and metabolic demands in the ischemic area. The aim of this review is to discuss recent changes in the field of the influence of BP and its management in AIS and to present the perspectives of a complex approach to this important aspect of AIS care.

## Pre-stroke Blood Pressure

Pre-stroke HT is reported in 30–80% of AIS patients and, according to the INTERSTROKE study, accounts for approximately 32 to 45% of the population-attributable risk of AIS. Moreover, hypertension has been shown to be the main contributor to stroke mortality [3]. The prevalence of HT is the highest among patients with large artery and small vessel pathologies and less common in patients with cardioembolic stroke [4]. It is noteworthy that the frequency of hypertension increases with age, regardless of the stroke subtype [4].

It is clear that the incidence and mortality in AIS are associated with BP ≥ 140/90 mmHg (hypertension), but the risk also increases when BP exceeds values as low as 115/75 mmHg, especially when such values occurred early in life (higher BP load). Indeed, even prehypertension infers a greater risk of stroke than optimal BP levels [5]. Thus, the thresholds for defining HT dichotomously as a risk factor for stroke should be reconsidered.

Although sustained hypertension clearly increases the incidence of stroke, also a transient and repetitive rise in BP, referred to as BP variability (BPV), both short-term (day-night) and long-term (seasonal or visit-to-visit) BPV are related to an increased risk of stroke [6]. Moreover, an abnormal BP circadian course, characterized by an insufficient nocturnal systolic BP (SBP) decrease (i.e., < 10%) compared to daily values, was proven to be associated with an increased risk of stroke [7]. Morning surge, characterized by an extreme SBP rise during the wake-up time, appears to be of particular relevance and represents subpopulations at an elevated risk of ischemic stroke and other stroke subtypes [8••].

Racial disparities in the burden of hypertension-related diseases may account for stroke incidence. African Americans as well as sub-Saharan Africans are known to have a higher prevalence of HT, nocturnal HT, and a non-dipping BP profile which starts earlier than in white people [9, 10]. They also have higher cardiovascular risk than white people, including ischemic stroke, which is largely driven by nocturnal BP [11].

A steeper association of BP with stroke among Asians than among European populations was also observed [12]. A similar pattern was reported in Latin American countries, with the mean patient age at stroke onset being approximately 10 years younger than in high-income countries (e.g., North America) [13].

Based on the aforementioned, it is highly probable that an excess risk of stroke, partially beyond pure office BP values, may exist in the majority of ethnic groups worldwide.

## Blood Pressure in Acute Stroke

In the acute phase of AIS, increased BP (often reflecting a transient reaction thus called acute hypertensive response, AHR) is observed in approximately 75% of patients [14]. AHR is usually at its highest within the first hours after stroke onset, then gradually declines [15], and usually settles within the first 7–10 days after stroke onset [16, 17].

Interestingly, in some patients (particularly younger ones), the absolute values of increased BP remain within “normotensive” limits. The mechanisms of this clinical setting are not clear and probably very heterogenous.

AHR is a phenomenon caused by a myriad of factors [18]. On one hand, it might be related to preexisting conditions, including inadequately treated or undiagnosed hypertension [19–22] or diabetes mellitus [23]. On the other hand, it may be linked to stroke-related factors such as damage to the brain regions involved in autonomic regulation [24] or secondary sympathetic adrenomedullary pathway activation due to the stroke itself [25–28].

Frequently, it is associated with AIS consequences, e.g., urine retention, infection, cerebral edema, headache [29, 30], or stress related to hospitalization [21, 31].

Early recanalization can be achieved in up to 30% of patients treated with IVT alone [32], and the rate may increase to even 80–90% [33, 34] in patients treated with MT. But even in a best-case scenario, only up to 50% of patients who receive this treatment may achieve functional independence, partially due to the fact that the core of the insult is already too large at the time of reperfusion [35]. Recently, however, it has been postulated that tissue outcome following AIS is associated with multiple factors and mechanisms, other than those directly related to local hypoperfusion, the potential outcome-based significance of which is illustrated by a recently described reverse mismatch phenomenon [36–38].

This partially depends on individual factors such as vascular compliance or collaterals [39]. The latter are crucial in maintaining CBF in the penumbra, distally from the occluded artery [40]. Collaterals are associated with lower infarct volume growth and thus with better functional outcome [41]. As it has been recently shown in patients undergoing endovascular treatment, the odds for a good clinical outcome were threefold higher in patients with good collaterals than in patients with absent collaterals [42]. However, collateral blood flow not only depends on individual anatomy but also on adaptive features like cerebral autoregulation (CA). Studies indicate that dynamic CA may be damaged by AIS, irrespective of the stroke subtype, for up to 96 h, and remain abnormal for at least 1–2 weeks after the stroke [43]. Previous studies on static CA showed that it may remain preserved in AIS [44]. In the case of dysregulated CA, CBF within the penumbra depends on systemic BP [45]. Within recent years, the relationship between blood pressure and collateral blood flow has been investigated. It is postulated that BP may influence collateral blood flow in AIS.

On the other hand, BP values in the acute phase of stroke may reflect the individual efficacy of local (collaterals) and systemic (volemia, cardiac output, vascular reaction) compensatory mechanisms.

Sufficient collateral blood flow results in slower [46] and smaller ischemic lesion growth [47].

The majority of studies indicate that higher acute phase BP is associated with better collaterals in ischemic stroke patients [48–52]. There is, however, some evidence from a few studies showing that lower BP may favor better pial collateral recruitment [53].

The majority of studies indicate that higher BP may be an important factor improving cerebral blood flow in the penumbra, however, under one major condition—reperfusion. A study by Hong et al. suggests that higher BP may result in a smaller infarct volume but only in patients with major reperfusion. In the case of reperfusion failure, sustained high BP may result in increased infarct growth and an unfavorable outcome [54••].

BP patterns may also influence outcomes even in patients with poor collaterals. Lower BP fluctuations after reperfusion were shown to be associated with better long-term outcomes in patients undergoing MT.

## Blood Pressure Management

### Pre-hospital Phase

The proper management in the pre-hospital phase is decisive in the treatment of ischemic stroke. As recanalization and reperfusion are crucial factors influencing long-term functional outcome [55], shortening of the onset-to-recanalization time should be the main aim of pre-hospital treatment. However, since recanalization may not result in a clinical improvement in up to half of patients [35], one should not neglect other potentially modifiable conditions, such as blood oxygenation, glucose level, body temperature, and BP. According to the current American Heart Association (AHA) guidelines [56••] and European Stroke Organization (ESO) Consensus Statements and Recommendations [57], both published in 2018, BP should not be lowered in the pre-hospital phase. This has been confirmed by recently published results of the RIGHT-2 trial [58••], which showed that in patients with a presumed stroke and elevated BP, pre-hospital antihypertensive treatment with transdermal glyceryl trinitrate (GTN) does not improve functional outcome. However, this study, together with previous smaller trials [59], showed that ambulance-based studies are feasible and needed. A new study assessing transdermal GTN in the hyperacute phase of ischemic stroke is currently ongoing [60].

### Intrahospital Phase

Both the AHA and ESO guidelines indicate that in patients who are not qualified for either IVT or MT, BP should not be lowered, unless it exceeds 220/120 mmHg. It is, however, highlighted that patients with AIS, presenting symptoms of other severe, acute comorbidities (e.g., acute coronary event, acute heart failure, aortic dissection, or preeclampsia/eclampsia), may require an emergency BP reduction. It is underlined that a BP reduction should be careful and individualized since an exaggerated drop in BP can result in complications, such as stroke progression or acute kidney injury [56••, 57]. According to the AHA, a reasonable goal is a 15% reduction in the initial BP [56••]. To date, no BP reduction strategy has been shown to be superior.

### Specific Treatment

#### Intravenous Thrombolysis

According to the current AHA and ESO guidelines, patients who are eligible for treatment with intravenous thrombolysis (IVT) should have their BP carefully lowered so that their SBP is < 185 mmHg and their diastolic BP (DBP) is < 110 mmHg before IVT is initiated and < 180/105 mmHg during the first 24 h after the treatment [56••, 57]. These thresholds are extrapolated from thrombolysis trials in myocardial infarction as well as from the National Institute of Neurologic Disorders (NINDS) t-PA Trial [61].

However, BP levels higher than those recommended are observed in up to 50% of patients who are eligible for IVT [62].

It has been retrospectively shown that higher BP significantly increased the risk of hemorrhagic transformation [61, 63]. The risk increased linearly, being four times higher in patients with SBP > 170 mmHg compared to those at 141–150 mmHg [64].

Moreover, it also increased in patients with higher BP variability [64–66]. This is in line with a finding that a cumulative decline in SBP > 50 mmHg or an acute drop of > 30 mmHg was associated with a decreased likelihood of a favorable outcome. It is noteworthy that an acute drop in SBP, DBP, or MBP > 60 mmHg was associated with an increased risk of death [67].

The exact BP at which the risk of hemorrhage after intravenous thrombolysis increases is rather individual and probably depends on BP per se but also on other acute and chronic factors [68••].

Recently, ENCHANTED, a randomized control trial (RCT) conducted in patients treated with IVT, showed a reduced risk of hemorrhagic transformation in patients treated intensively (target systolic blood pressure at 130–140 mmHg) when compared to patients treated according to the guidelines over the first 72 h after stroke onset. This, however, did not translate into an improved clinical outcome [68••].

In a recent meta-analysis, Malhotra et al. evaluated the impact of pre- and postthrombolysis BP values on clinical outcome in over 56,000 AIS patients. In this group, a higher pre- and posttreatment SBP was associated with a higher risk of symptomatic intracerebral hemorrhage (sICH).

Moreover, elevated pre- and postinfusion BP was associated with worse long-term functional outcome, even after adjustment for potential confounders [69•].

Another interesting finding of Malhotra’s meta-analysis is that higher mean pre-treatment SBP levels were recorded in AIS patients with proximal intracranial occlusion who did not achieve tissue plasminogen activator-induced recanalization. This suggests that elevated SBP might be a surrogate of increased baseline thrombus burden or impaired endogenous fibrinolysis [69•].

#### Mechanical Thrombectomy

Since 2015, mechanical thrombectomy has been a standard of treatment for ischemic stroke patients with anterior circulation large artery occlusions. In patients undergoing MT, and who have not received IVT, both the AHA and ESO recommend maintaining BP at ≤ 185/110 mmHg before the procedure, as it was in 5 of 6 RCTs that demonstrated clinical benefits from MT with stent retrievers up to 6 h from stroke onset (REVASCAT [70], SWIFT PRIME [71], EXTEND-IA [72], THRACE [73], and MR CLEAN [74]) [56••, 57]. It is suggested to maintain BP at ≤ 180/105 mmHg during, and for the 14 h after, the procedure, regardless of whether or not the recanalization has been achieved [56••, 57]. These guidelines, however, are not based on strong, scientifically proven data. There are, however, several new studies which have addressed the problem of BP treatment on functional outcome in patients undergoing MT. It has been shown that either pre-, intra-, and postprocedural low and high BP and BP fluctuations may negatively influence outcome in this group.

The post hoc analysis of MR CLEAN demonstrated a U-shaped relationship between baseline SBP and poor functional outcome. The most favorable BP was 120 mmHg, and a 21% increase in the relative risk of hemorrhage for every 10 mmHg above this value was shown [75].

This is in line with an analysis of baseline BPs from over 1300 patients enrolled in the Endovascular Treatment in Ischemic Stroke registry. In this group, there was a higher risk of mortality of 3.78 and 1.81 times for SBP < 110 mmHg and > 180 mmHg, respectively, compared with SBP 150–160 mmHg as a reference [76–78].

Intraprocedural BP is another important hemodynamic factor influencing outcome in patients undergoing MT. In a large study, patients with a favorable outcome presented moderately elevated SBP (average maximal SBP 164 mmHg) compared with those with an unfavorable outcome (average maximal SBP 181 mmHg) [79]. On the other hand, intraprocedural hypotension is a strong predictor of poor outcome. In a study by Lowhagen Henden et al., a mean BP (MBP) fall of > 40% from the baseline was an independent predictor of an unfavorable neurological outcome, in addition to well-established risk factors such as a high baseline NIH Stroke Scale (NIHSS) or a lack of successful recanalization [80]. The results have been confirmed by more recent studies [81, 82].

One of the most important factors influencing blood pressure levels during the MT procedure is anesthesia. In recent years, conscious sedation (CS) has been preferred over general anesthesia (GA) as it was shown to be related with better functional outcome and decreased mortality [83]. This was mainly related to the effect GA may have on hemodynamics. Among MR CLEAN patients, the use of GA was associated with larger drops in MBP and longer episodes of hypotension [84]. However, CS also carries a risk of hypotension. In a study by Wahlin et al., among patients treated with MT under CS, an MBP drop of ≥ 10% was associated with the highest risk of poor outcome. MBP < 85 mmHg before reperfusion, and every 10 mmHg a drop in mean arterial pressure to below 100 mmHg were also associated with a worse prognosis [85].

Recent data from prospective trials can contribute to a change in everyday practice. The GOLIATH, SIESTA, and AnSTROKE studies showed no differences in the clinical effect, regardless of the type of anesthesia. Although GA was associated with more frequent drops in MBP > 20% from the baseline compared to CS, there was no significant difference in large falls in MBP (either defined as a decline of > 40% or MBP < 70 mmHg) between GA and CS [86•, 87•, 88]. These results are important evidence that CS and GA can be equivalent methods of anesthesia in patients undergoing mechanical thrombectomy.

BP treatment in the post MT period is another important issue. Recanalization is achieved in up to 70–80% of patients [33, 34], which in a large portion of patients leads to a spontaneous reduction of BP. In those in which BP remains elevated, it increases the risk of hemorrhagic transformation and therefore poor functional outcome. In a study by Goyal et al., patients with intensively (< 140/90 mmHg) and moderately (< 160/90 mmHg) lowered BP levels experienced lower mortality rates when compared to patients with permissive BP levels (< 220/120 mmHg or < 180/110 mmHg if IVT was administered) [78]. This is in line with the finding that higher peak values of SBP in the first 24 h after MT are associated with worse functional outcomes at 90 days and greater severity of hemorrhagic complications within 48 h after MT [89].

The studies in the field of BP management during MT have been summarized in a recent systematic review by Maier et al. The results indicate particularly that BP drops during the procedure may be associated with poor functional outcome [90••]. The data show that in the studies where the BP level was strictly maintained between 120 (140) and 180 mmHg, there was no association between the BP level and the outcome [91, 92].

## Low Blood Pressure in AIS

Low BP levels are detected in about 5–10% of patients with ischemic stroke, and this was shown to be related with poor functional outcome [93, 94]. Of note, the etiology of AIS presenting low BP might be unrelated to occlusive vessel disease, but this is beyond the scope of the article.

Low BP may be triggered by conditions other than neurological ones, such as hypovolemia [95], myocardial infarction, heart failure [94], takotsubo syndrome, cardiac arrhythmia, aortic dissection, and blood loss, including retroperitoneal hemorrhage and peri-procedural hemorrhage during MT. A significant BP drop may occur during the induction phase of GA during MT, since a drop in BP is a side effect of most anesthetic medications [96]. However, not only GA but also CS causes a decline in BP [97, 98] (see above).

According to the latest AHA/ASA recommendations, hypotension and hypovolemia should be corrected to maintain a systemic perfusion level necessary to support organ function [56••]. It is not indicated whether colloids or crystalloids should be used. The authors, however, do not indicate the BP level below which the treatment should be initiated, how long it should be maintained, and which BP level should be a treatment goal.

One should, however, remember that excessive fluid therapy may exacerbate chronic diseases like anemia and eventually result in ischemic lesion growth [99].

Recently, Bang et al. published the results of a randomized study, evaluating the impact of phenylephrine-induced hypertension on short- and long-term outcome in patients with non-cardioembolic stroke, causing a major neurologic deficit (baseline NIHSS score 4–18 points), who were ineligible for recanalization therapy (IVT or MT) or who experienced a progression of the stroke. Patients with SBP > 170 mmHg were excluded from the study. Induced hypertension was associated with early neurologic improvement and functional independence at 90 days, without severe complications [100••]. This is the first randomized trial in this field, which may contribute to a change in future guidelines.

## BP Management in Acute Ischemic Stroke Patients With and Without Hypertension

In contrast to BP values, it is speculated that the history of hypertension may serve as a reliable predictor of stroke outcome. The presence of hypertension and related end organ damage should be accounted for in the individual overall cardiovascular risk, in general, as important determinants of both vascular and overall health.

Indeed, AIS patients with premorbid HT have an increased risk of cerebral hypoperfusion or edema with a decline or rise in BP, respectively. Moreover, patients with AIS and premorbid HT have a better prognosis at higher admission BP values compared with those with preexisting normotonia [101].

Recent data have also suggested that the results of BP reductions in AIS should be adjusted for premorbid hypertension.

In a Chinese trial, CATIS (China Antihypertensive Trial in Acute Ischemic Stroke), conducted among 4071 patients with non-thrombolyzed ischemic stroke within 48 h of onset and elevated systolic blood pressure, it was found that early antihypertensive treatment was not associated with a combined primary outcome, defined as death or significant disability (modified Rankin Scale score ≥ 3 > 2 points), at day 14 or hospital discharge, among patients with or without hypertension. However, a history of premorbid hypertension appeared to be discriminative regarding secondary stroke prevention. In patients with pre-stroke hypertension, early antihypertensive treatment was associated with a lower rate of 3-month recurrent strokes. Conversely, non-hypertensive patients treated with antihypertensives in the initial stages of AIS tended to have more recurrent strokes (*p* = 0.06) [102••]. Of note, the results of the CATIS trial should be interpreted with caution, because they may not be valid for other populations than the Chinese, where the prevalence of hypertension is high, and stroke is more common than coronary heart disease, especially in rural areas [103].

Hypertension may also modify the clinical outcome in patients undergoing a specific treatment of AIS. Available evidence shows that arterial hypertension is related to poor functional outcome after intravenous thrombolysis [104]. However, in a recently published multicentre study of ischemic stroke in patients eligible for intravenous thrombolysis (ENCHANTED study), hypertension favored neither guideline-recommended blood pressure lowering nor intensive blood pressure lowering [68••]. Importantly, this trial excluded patients with SBP > 185 mmHg.

In a recent meta-analysis on the effects of hypertension in patients undergoing mechanical thrombectomy (MT), it was shown that the presence of hypertension was associated with a lower rate of 90-day independence, regardless of the stroke severity. However, this relationship was observed in Europeans, but not in Asian and American populations. Furthermore, hypertensive patients had also a higher rate of mortality after 3 months. Interestingly, there were no associations between hypertension status and symptomatic intracerebral hemorrhage in patients with acute ischemic stroke treated with MT [105].

In summary, HT is not only one of the most important risk factors of ischemic stroke but also it may have an influence on functional outcome. However, whether it is necessary to have different BP management strategies for patients with acute ischemic stroke with or without a history of hypertension before stroke onset is unclear.

## Future Perspectives

### Other Hemodynamic Parameters in Ischemic Stroke

Extensive research demonstrates that arterial stiffening (reduced compliance and distensibility of arterial walls due to their structural and functional alterations, with a resultant rise in pulse wave velocity (PWV), along with the premature return of reflected pressure waves to the heart, excessive augmentation of central BP, and the transmission of flow pulsations to the periphery) has emerged as one of the earliest manifestations of vascular disease [106].

Arterial stiffness is a potent risk factor for cardiovascular complications, including stroke, in the same way as hypertension, and irrespective of BP levels.

We and other authors showed that higher PWV is associated with a worse early and late clinical outcome after ischemic stroke [107–112]. Increased PWV was also found to be associated with a higher risk of vascular death [113]. Furthermore, our team demonstrated that acute hypertensive response, the most typical hemodynamic consequence of AIS, is independently associated with increased aortic stiffness [114].

Another of our studies revealed that preserved left ventricular ejection fraction and aortic compliance are associated, independently of each other, with better neurologic outcome 10 days after ischemic stroke onset; the worst outcome was observed in patients with both stiff aorta and low ejection fraction [112].

The central augmentation index (cAIx) is a marker indirectly associated with arterial stiffness and dependent on other cardiovascular factors, such as heart rate, systolic ejection period, and peripheral vasoconstriction. Central pressure is the main drive for target organs, and therefore it seems to be a more reliable haemodynamic parameter than peripheral BP, both in physiology and pathology.

Studies on the association between cAIx and outcome after ischemic stroke are conflicting, as are studies on the relationship between central pressure parameters and cardiovascular events. Recently, we showed that a rise in the augmentation index between days 1 and 6 after stroke onset is associated with a better both early and late functional status [115]. The significance of central BP parameters may also be of great interest and still may not be fully understood, requiring further research.

Of note, BP, as one of the key components of the complex hemodynamic response during ischemic stroke, is varied and inter-personally individualized according to acute and chronic mechanisms attempting to counteract the blood compromise in the ischemic brain area. Therefore, the individualization of management protocols, depending on a more complex evaluation of cerebral and global circulation, may be important in the future.

### Neuromonitoring: Dynamic and Personalized Hemodynamic Control

Recently, local and global intracranial parameters, including abnormal patterns of cerebral blood flow in cerebral large arteries or increased intracranial pressure, as assessed with transcranial Doppler (TCD), have been found to be predictive of tissue recovery and functional outcome after ischemic stroke [116]. In a small Chinese RCT study, adequate TCD-guided BP control improved the prognosis of patients with AIS in the anterior circulation who underwent MT. For those who presented ultrasonographic signs of blood flow deceleration or features of intracranial hypertension, BP manipulation under the guidance of TCD monitoring appeared to reduce early neurological deterioration and improved the final functional outcome.

Similarly, dynamic instead of fixed BP thresholds have been postulated as a guide for individualizing hemodynamic management in AIS. In a small observational study from the USA, Petersen et al. proposed a novel approach to define the limits of autoregulation (LA) using near-infrared spectroscopy (NIRS). Continuous non-invasive NIRS neuromonitoring in response to changes in mean arterial pressure was found to identify and track the patient-specific BP range at which autoregulation was optimally functioning in individual patients after large-vessel ischemic stroke [117].

Data suggests that LA values are not stable but change over time and vary among individuals with AIS. Exceeding these individual and flexible thresholds of autoregulation was associated with worse functional outcome compared to the BP range within LA. An increased risk of sICH and neurological worsening was associated with elevated BP relative to each patient’s personalized upper LA, rather than the absolute increased BP alone [118].

This novel approach to define personalized, autoregulation-based BP thresholds in acute ischemic stroke may present a better treatment strategy as compared with traditional BP management, which aims to maintain BP below the fixed thresholds, but therapies based on patients’ autoregulatory statuses need further research in randomized trials.

### Complex Cardiopulmonary Monitoring: Confounders Influencing Bp and Outcome

Finally, traditional monitoring parameters (heart rate, BP, oxygen saturation) are secondary to many compensatory mechanisms, depending on the patient and stroke baseline characteristics.

Approximately 25% of AIS are of cardiac origin [119]. Cardiac dysfunction can worsen the pre-stroke cerebral damage. An adequate cardiac output (CO) corresponds to adequate oxygen delivery. Recently, CO has been shown to be better associated with cerebral perfusion than blood pressure in ischemic stroke and with stroke outcome [120]. Indeed, cardiovascular complications, including acute on-chronic coronary syndrome, arrhythmias, autonomic dysfunction, and takotsubo syndrome, with or without subsequent heart failure, are observed in the majority of AIS patients within the first 24 h [121]. A novel study found that multimodal brain imaging performed in acute stroke may accurately predict reduced left ventricle ejection fraction (LVEF). The arterial input function width on perfusion computed tomography has been shown to correlate with LVEF in AIS and to identify patients with a reduced LVEF and increased risk of worse clinical outcome 3 months after the event [122].

Furthermore**,** obstructive sleep apnea (OSA), a disorder characterized by recurrent episodes of upper respiratory tract obstruction during sleep, accompanied by intermittent hypoxia, is very common in AIS patients, with a prevalence of 61.9%. Of importance, patients with AIS and OSA have been shown to demonstrate a higher SBP after an ictus, as well as a higher rate of cardiac alterations, including an increased left ventricular wall thickness and left atrial diameter [123]. Both OSA and cardiac damage play a crucial role in AIS.

Thus, metabolic and hemodynamic optimization, both at the local and global level, may need to be considered as a potent neuroprotective strategy, though RCTs are needed to determine the optimal hemodynamic approach.

## Conclusions

AIS is a major medical emergency with heterogenous pathophysiology across patients and over time. Elevated BP is the most common clinical symptom recorded at stroke presentation; however, low BP may also occur, yet much less frequently.

The physiological basis for the abrupt changes in BP shortly after focal cerebral ischemia remains poorly understood. Such changes represent many mechanisms, both nonspecific and stroke related, that resolve within hours and days in the vast majority of subjects, but it remains debatable as to whether they are of therapeutic relevance.

Recent data show the importance not only of pre-recanalization but also intra- and postprocedural BP and its variability.

However, measures other than BP, including stroke and patient characteristics, may need to be considered in the early evaluation of the prognosis and treatment of altered BP in AIS.

Individual parameters of cerebral circulation, such as autoregulation-derived, personalized BP range, as well as the broader aspects of hemodynamic homeostasis (cardiac output, pulse wave velocity) have also been postulated as more robust and reliable predictors of functional outcome in patients with AIS than BP alone. Advanced cerebral and systemic hemodynamic monitoring might be valuable goals in order to optimize the penumbral perfusion in AIS.
